# Evaluating the psychometric properties of the simplified Chinese version of PROMIS-29 version 2.1 in patients with hematologic malignancies

**DOI:** 10.1038/s41598-024-61835-4

**Published:** 2024-05-15

**Authors:** Qianqian Zhang, Jinying Zhao, Yating Liu, Yan Cui, Wen Wang, Junjie Li, Yanxia Liu, Fei Tian, Zhixin Wang, Huijuan Zhang, Guiying Liu, Yun Wu, Qiuhuan Li, Tingyu Hu, Wen Zhang, Wenjun Xie

**Affiliations:** 1 State Key Laboratory of Experimental Hematology, National Clinical Research Center for Blood Diseases, Haihe Laboratory of Cell Ecosystem, Institute of Hematology and Blood Diseases Hospital, Chinese Academy of Medical Sciences and Peking Union Medical College, Tianjin, 300020 China; 2 Tianjin Institutes of Health Science, Tianjin, 301600 China; 3 The First Affiliated Hospital of the University of Science and Technology of China (Anhui Provincial Hospital), Anhui, 230001 China; 4 Qilu Hospital of Shangdong University, Shandong, 250012 China; 5 The Affiliated Hospital of Xuzhou Medical University, Jiangsu, 221002 China; 6 School of Nursing, Fudan University, 305 Fenglin Road, Shanghai, 200032 China

**Keywords:** Psychometric evaluation, PROMIS-29, Hematological malignancy, Patient-reported outcomes, Cancer, Psychology

## Abstract

The Patient-Reported Outcomes Measurement Information System 29-item Profile version 2.1 (PROMIS-29 V2.1) is a widely utilized self-reported instrument for assessing health outcomes from the patients’ perspectives. This study aimed to evaluate the psychometric properties of the PROMIS-29 V2.1 Chinese version among patients with hematological malignancy. Conducted as a cross-sectional, this research was approved by the Medical Ethical Committee of the Institute of Hematology and Blood Diseases Hospital, Chinese Academy of Medical Sciences and Peking Union Medical College (registration number QTJC2022002-EC-1). We employed convenience sampling to enroll eligible patients with hematological malignancy from four tertiary hospitals in Tianjin, Shandong, Jiangsu, and Anhui province in China between June and August 2023. Participants were asked to complete a socio-demographic information questionnaire, the PROMIS-29 V2.1, and the Functional Assessment of Cancer Therapy-General (FACT-G). We assessed the reliability, ceiling and floor effects, structural, convergent discriminant and criterion validity of the PROMIS-29 V2.1. A total of 354 patients with a mean age of 46.93 years was included in the final analysis. The reliability of the PROMIS-29 V2.1 was affirmed, with Cronbach’s α for the domains ranging from 0.787 to 0.968. Except sleep disturbance, the other six domains had ceiling effects, which were seen on physical function (26.0%), anxiety (37.0%), depression (40.4%), fatigue (18.4%), social roles (18.9%) and pain interference (43.2%), respectively. Criterion validity was supported by significant correlations between the PROMIS-29 V2.1 and FACT-G scores, as determined by the Spearman correlation test (P < 0.001). Confirmatory factor analysis (CFA) indicated a good model fit, with indices of χ^2^/df (2.602), IFI (0.960), and RMSEA (0.067). The Average Variance Extracted (AVE) values for the seven dimensions of PROMIS-29 V2.1, ranging from 0.500 to 0.910, demonstrated satisfactory convergent validity. Discriminant validity was confirmed by ideal √AVE values. The Chinese version of the PROMIS-29 V2.1 profile has been validated as an effective instrument for assessing symptoms and functions in patients with hematological malignancy, underscoring its reliability and applicability in this specific patient group.

## Introduction

Hematological Malignancy (HM) represents a complex group of highly malignant tumor diseases that are challenging to treat. According to 2020 WHO statistics, the incidence rates of leukemia in China was 5.9 per 100,000, non-Hodgkin lymphoma, multiple myeloma and Hodgkin lymphoma were 6.4 per 100,000, 0.47 per 100,000, 1.5 per 100,000 respectively^1^. Patients afflicted with HM often grapple with a myriad of physical, psychological, and social challenges, exacerbated by both the disease and its associated treatments. In the realm of cancer care, the frequent and precise assessment of symptoms is paramount. Patient-reported outcome (PRO) measures have emerged as a gold standard, offering invaluable insights into patients’ subjective experiences and overall quality of life^2^. These tools are instrumental in fostering enhanced patient-nurse communication, enabling systematic monitoring, and facilitating tailored management of patients’ symptoms, thereby promoting patient-centered care^3–6^.

The Patient-Reported Outcomes Measurement Information System (PROMIS), an initiative by the National Institutes of Health, is renowned for its innovative self-report measures designed to evaluate the physical, mental, and social facets of health and well-being^7^. The versatility and comprehensiveness of PROMIS have garnered significant attention, marking it as a pivotal tool in the holistic assessment of individual health^2,8,9^. PROMIS includes item banks that can be administered using computer-adaptive testing, short forms for individual domains, and profiles that yield information about multiple domains for use in clinical trials, observational studies, and clinical practice^7^.

The PROMIS-29 V2.1, in particular, stands out for its robust design, aimed at addressing the gap in universal and generalizable measures for assessing core patient-reported symptoms and functional domains in individuals with chronic diseases^10^. Developed through meticulous processes including literature review, Item Response Theory (IRT) analysis, and expert reviews, the PROMIS-29 V2.1 ensures a comprehensive and standardized evaluation of patients’ health statuses^10^.

Although the PROMIS-29 V2.1 has been translated into Chinese by the PROMIS National Center-China (PNC-China), its application in the context of HM remains limited. There is a conspicuous absence of validation studies exploring the efficacy and reliability of PROMIS-29 V2.1 among HM patients. Given the critical need for nuanced assessments of physical, social, and mental health in this demographic, validating the PROMIS-29 V2.1 could not only enhance clinical practices but also pave the way for international comparative studies.

In light of this, our study is poised to conduct an exhaustive psychometric evaluation of the Chinese version of PROMIS-29 V2.1 among a selected cohort of HM patients in mainland China. We aim to delineate its reliability, validity, and potential applications in this specific medical and cultural context.

## Methods

### Study design

This multicenter cross-sectional study received approval from the Medical Ethical Committee of Institute of Hematology and Blood Diseases Hospital, Chinese Academy of Medical Sciences and Peking Union Medical College (registration number QTJC2022002-EC-1). We adhered to the Consensus-based standards for the selection of health measurement instruments (COSMIN) guidelines to evaluate the psychometric properties of the Chinese version of the PROMIS-29 V2.1 among hematological malignancy patients.

### Setting and sample

Patients were conveniently sampled from the hematology departments of four tertiary hospitals across Tianjin, Shandong, Jiangsu, and Anhui provinces in China, between June and August 2023. Based on the 5–10:1 case-to-variable ratio for psychometric evaluation and accounting for a potential 20% invalid sample rate, we aimed for a sample size between 174 and 348 and successfully included 354 cases^9,11^. The sample size was aslo sufficient to perform stable and precise model estimation by confirmatory factor analysis (CFA)^11^.

Patients were eligible for the study if they met the following criteria: (a) aged 18 or older, (b) had a diagnosis of Hematological Malignancy, including leukemia, lymphoma, myeloma, myelodysplastic neoplasms and myeloproliferative neoplasms, (c) Being able to speak Mandarin and read Chinese, and (d) signed an informed consent form. Patients with psychiatric illness, cognitive impairment or diagnosis of another cancer type were excluded.

### Measures

#### Socio-demographic information questionnaire

A sociodemographic information questionnaire was developed to collect sociodemographic and clinical data including gender, age, residential location, education level, marital status, job, employment status, health insurance, average monthly family income, primary caregiver, diagnose, time since diagnosis, medical costs, treatment phase and medical treatment. Patients self-reported sociodemographic data, while trained nurse researchers extracted clinical data from medical records.

#### PROMIS-29 V2.1

The Chinese version of the PROMIS-29 V2.1 was used in this study, which was authorized by PNC-China. The PROMIS-29 V2.1 consists of 29 items measuring seven health and function domains: physical function, anxiety, depression, fatigue, sleep disturbance, ability to participate in social roles and activities, pain interference and intensity. Except for a single item for pain intensity, all domains include 4 items and are responded to with a five-point Likert scale from 1 to 5. The pain intensity item is answered with a 0 to 10 numeric rating scale ranging from 0 (without pain) to 10 (worst pain imaginable). Item scores in each domain were summed and transformed into T-scores metric: values of 50 (SD = 10) indicate the mean of the U.S. general population (http://www.healthmeasures.net)^7^. For physical function and social role, higher scores indicate better functioning and quality of life (QOL). For depression, anxiety, fatigue, pain interference, pain intensity, and sleep disturbance, a higher score indicates more serious implications of disease^7^.

#### FACT-G

The FACT-G are the most frequently used questionnaires to measure health-related quality of life (HRQOL) in patients with cancer. The FACT-G is comprised of four subscales: physical wellbeing (PWB, 7 items, 0–28), social/family wellbeing (SWB, 7 items, 0–28), emotional wellbeing (EWB, 6 items, 0–24), and functional wellbeing (FWB, 7 items, 0–28)^12^. All items in the FACT-G use a five-point rating scale (0 = not at all, 1 = a little bit, 2 = somewhat, 3 = quite a bit, and 4 = very much). The 12 items PWB l to 7, EWB l, EWB 3 to EWB 6 are reverse entries and need to be scored in reverse. The total score of the scale is 108, and the higher the score, the higher the quality of life^12,13^.

### Date collection

Eligible patients were enrolled during hospitalization by the trained nurse researchers at each study site, who had received training regarding the study process to ensure the standardization of the data collection. All the participants were informed about the purpose and procedures of the study, and verbal consent was obtained before data collection. In addition, participants were informed of the voluntary nature of participation, participants’ rights, and the confidentiality of the data. Participants could choose to complete the survey either on paper or using web-based questionnaires based on their preferences. Data on every respondent were collected only once. The participants were required to return the questionnaire immediately after completion. To express gratitude, all participants were distributed a bottle of no-hand sanitizer after completing the questionnaire.

### Date analysis

Analyses were conducted using IBM SPSS version 21.0 and IBM SPSS Amos Graphics (version 26.0). All significance tests were 2-tailed, with p < 0.05 considered signifcant.

Descriptive statistics were calculated for sample characteristics and study variables, in which continuous variables were analyzed by means and standard deviations, and categorical variables were described by counts and percentages. The PROMIS-29 V2.1 raw scores were transformed into T-scores based on the PROMIS guidelines (http://www.healthmeasures.net). The ceiling or floor effects were identified if responses exceeded 15% at the best and the worst possible score. Reliability was assessed via Cronbach’s α coefficient, Composite Reliability (CR) and split-half reliability.

Criterion validity was determined by correlating PROMIS-29 V2.1 domains with similar constructs in FACT-G, using Spearman correlation coefficients. Confirmatory factor analysis (CFA) was carried out using maximum likelihood estimation to examine the construct validity of the PROMIS-29 V2.1 domains. To examine the goodness of model fit, indices including the χ^2^/degree of freedom (χ^2^/df), root mean square error of approximation (RMSEA), goodness-of-fit index (GFI), comparative fit index (CFI), incremental fit index (IFI), Normed Fit Index (NFI), and Tucker–Lewis index (TLI) were included. An acceptable CFA model should have a χ^2^/df < 3; a RMSEA < 0.08; and a GFI, CFI, IFI, NFI, TLI > 0.9^14^. AVE and √AVE index were performed to assess the convergent validity and discriminant validity.

### Ethics approval and consent to participate

All participants signed written informed-consent forms and completed questionnaires online at their earliest convenience. Ethical approval was approved by the Institute of Hematology and Blood Diseases Hospital, Chinese Academy of Medical Sciences and Peking Union Medical College (No. QTJC2022002-EC-1).

## Results

### Sample characteristics

A total of 400 questionnaires were distributed. 29 eligible participants did not consent to participate, while 371 agreed to be involved. In addition, 17 questionnaires were excluded for that the participants circled the same response choice for every question asked. A sample of 354 was chosen for the final analysis. The average age of the patients was 46.93 years. A majority of the participants were male (57.3%), married (78.8%), and unemployed (78.2%). In terms of education, the largest group had completed high school or an equivalent level of education (39.5%). Most participants were covered by employee health insurance (60.5%), and the prevalent income bracket was ¥3001–¥5000 per month (25.7%). Clinically, leukemia was the most common diagnosis, accounting for 43.8% of the patients. A significant portion (32.8%) were diagnosed for less than 6 months. The majority (83.6%) were undergoing treatment at the time of the survey. See more detail in Table 1.

**Table 1:** Sample characteristics of the study sample (N = 354).

Characteristic	
Gender [n (%)]	
Male	203 (57.3)
Female	151 (42.7)
Age (mean ± SD)	46.93 ± 16.15
Residential location [n (%)]	
Country	143 (40.4)
Town	88 (24.9)
Urban	123 (34.7)
Education level [n (%)]	
Primary school or below	36 (10.2)
Secondary school	109 (30.8)
High school or equivalent	140 (39.5)
University or above	69 (19.5)
Marital status [n (%)]	
Married	279 (78.8)
Single, divorced, widowed	75 (21.2)
Job [n (%)]	
Farmer	98 (27.7)
Staff	93 (26.3)
Retirement	72 (20.3)
Others	91 (25.7)
Employment status [n (%)]	
Employed	77 (21.8)
Unemployed	277 (78.2)
Health insurance [n (%)]	
Urban medical insurance	214 (60.5)
Rural health insurance	122 (34.5)
Self-paying	13 (3.6)
Commercial insurance	5 (1.4)
Average monthly family income [n (%)]	
< ¥1500	83 (23.4)
¥1501–¥2000	50 (14.1))
¥2001–¥3000	67 (18.9)
¥3001–¥5000	91 (25.7)
> ¥5001	63 (17.8))
Primary caregiver [n (%)]	
Parents	87 (24.6)
Sons or daughters	73 (20.6)
Spouse	179 (50.6)
Nursing worker	2 (0.6)
Someone else	13 (3.7)
Diagnose [n (%)]	
Leukemia	155 (43.8))
Lymphoma	99 (28.0)
Myeloma	49 (13.8)
Myelodysplastic neoplasms	40 (11.3)
Myeloproliferative neoplasms	2 (0.6)
Others	9 (2.5)
Time since diagnosis [n (%)]	
Less than half a year	116 (32.8)
Six months to a year	107 (30.2)
1 to 5 years	112 (31.6)
5 to 10 years	14 (4.0)
≥ 10 years	5 (1.4)
Medical costs (after insurance reimbursement) (mean ± SD)	¥298,880.7 ± ¥351,18.9
Treatment phase	
Pre-treatment	19 (5.4)
Under treatment	296 (83.6)
Post-treatment	39 (11.0)
Therapeutic regimen and medication category [n (%)]	
≤ 1 category	182 (51.4)
2–3 category	155 (43.8)
4–6 category	17 (4.8)

### Reliability analysis

Regarding the reliability analysis, the internal consistency coefficients, CR and split-half coefficient were calculated. Reliability was excellent for the PROMIS-29 V2.1 scale with Cronbach’s α (0.965) and split-half coefficient (0.927). For all seven domains of PROMIS-29 V2.1 subscales, Cronbach’s α ranged from 0.787 (sleep disturbance) to 0.968 (pain interference and intensity), CR ranged from 0.778 (sleep disturbance) to 0.976 (pain interference and intensity), which were all above the threshold of 0.70, indicating sufficient reliability. See Tables 2 and 6.

**Table 2:** Reliability of the PROMIS-29 V2.1 (N = 354).

Domain (items)	Cronbach’s α	Split-half coefficient
Physical function (4)	0.953	0.937
Anxiety (4)	0.964	0.941
Depression (4)	0.966	0.970
Fatigue (4)	0.966	0.953
Sleep disturbance (4)	0.787	0.584
Ability to participate in social roles and activities (4)	0.958	0.955
pain interference and intensity (5)	0.968	0.951
PROMIS-29 V2.1 (29)	0.965	0.927

### Descriptive statistics, ceiling, and floor statistics

Regarding the mean T-scores of PROMIS-29 V2.1, except the physical function (41.31 ± 11.85) and the ability to participate in social roles and activities (47.64 ± 11.38), the other five domains scores were significantly above than the reference level according to the PROMIS guidelines (http://www.healthmeasures.net). See Table 3.

**Table 3:** Mean Scores, T-scores, floor and ceiling effects of the PROMIS-29 V2.1 (N = 354).

Domain	Mean scores (mean ± SD )	T-scores (mean ± SD )	Lowest score [n (%)]	Highest score [n (%)]
Physical function	13.79 ± 5.89	41.31 ± 11.85*	4 [52 (14.7%)]	20 [92 **(26%)**]
Anxiety	8.65 ± 4.82	54.19 ± 12.66	4 [131 **(37.0%)**]	20 [19 (5.4%)]
Depression	8.26 ± 4.88	52.98 ± 11.77	4 [143 **(40.4%)**]	20 [17 (4.8%)]
Fatigue	10.44 ± 5.16	52.47 ± 12.85	4 [65 **(18.4%)**]	20 [33 (9.3%)]
Sleep disturbance	10.60 ± 3.87	50.75 ± 9.24	4 [35 (9.9%)]	20 [9 (2.5%)]
Ability to participate in social roles and activities	13.33 ± 5.23	47.64 ± 11.38*	4 [38 (10.7%)]	20 [67 **(18.9%)**]
pain interference	8.47 ± 5.13	52.77 ± 11.08	4 [53 **(43.2%)**]	20 [21 (5.9%)]

*Not meets cutoff.

Bold = domains with ceiling effects. For depression, anxiety, fatigue, pain interference, pain intensity, and sleep disturbance, if lowest score (%) exceeded 15%, they had ceiling effects; For physical function and social role, if highest score (%) exceeded 15%, they had ceiling effects.

Floor effects reflect the percentage of people who report the worst possible score; ceiling effects reflect the percentage of people who report the best possible score. And the ceiling or floor effects were identified if responses exceeded 15% at the best and the worst possible score. As mentioned in the methods section, for physical function and social role, higher scores indicate better functioning and QOL. For depression, anxiety, fatigue, pain interference, pain intensity, and sleep disturbance, a higher score indicates more serious implications of disease. As shown in Table 3, except sleep disturbance, the other six domains had ceiling effects, which were seen on physical function (26.0%), anxiety (37.0%), depression (40.4%), fatigue (18.4%), social roles (18.9%) and pain interference (43.2%), respectively. See Table 3.

### Criterion validity

After normality test, the scores of PROMlS-29 and FACT-G scale did not conform to normal distribution, so Spearman correlation analysis was used to conduct correlation analysis. The absolute value of correlation coefficient between PROMIS-29 V2.1 item scores with the corresponding domains coefficients in the FACT-G ranged from 0.156–0.752 (p < 0.001), showing satisfactory criterion validity. See Table 4.

**Table 4:** The criterion validity of the PROMIS-29 V2.1

Domain		FACT-G				
		Physical wellbeing	Social/family wellbeing	Emotional wellbeing	Functional wellbeing	FACT-G
PROMIS-29 V2.1	Physical function	0.652**	0.156**	0.379**	0.524**	0.544**
	Anxiety	− 0.633**	− 0.299**	− 0.698**	− 0.520**	− 0.665**
	Depression	− 0.601**	− 0.378**	− 0.716**	− 0.589**	− 0.701**
	Fatigue	− 0.752**	− 0.322**	− 0.593**	− 0.590**	− 0.692**
	Sleep disturbance	− 0.482**	− 0.357**	− 0.516**	− 0.522**	− 0.576**
	Ability to participate in social roles and activities	0.699**	0.314**	0.565**	0.586**	0.660**
	pain interference	− 0.677**	− 0.348**	− 0.540**	− 0.516**	− 0.652**
	PROMIS-29 V2.1	− 0.581**	− 0.377**	− 0.689**	− 0.520**	− 0.672**

**P < 0.001.

### Construct validity

**Figure 1:**
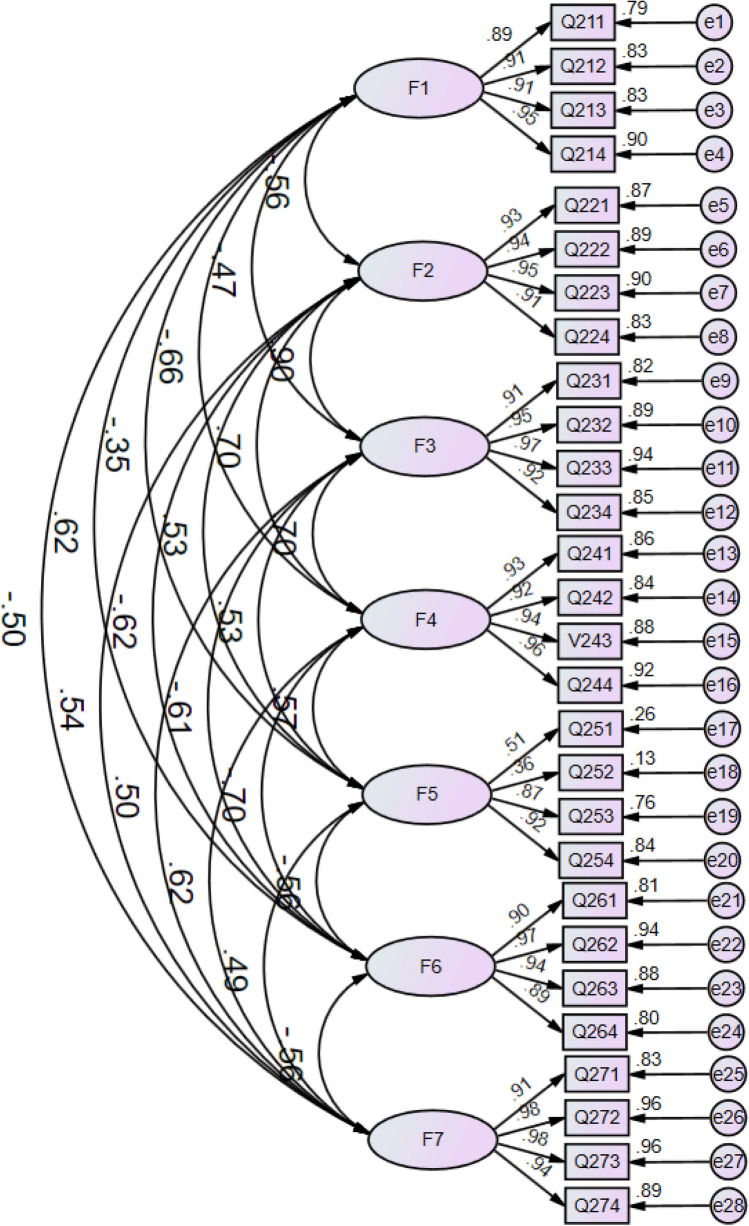
Confirmatory factor analysis model for PROMIS-29 V2.1 (F1–F7: anxiety, depression, physical function, fatigue, sleep disturbance, ability to participate in social roles and activities, and pain interference, respectively).

In our analysis, the PROMIS-29 V2.1 demonstrated excellent construct validity among patients with HM, as evidenced by a χ^2^/df of 2.602, an IFI of 0.960, and an RMSEA of 0.067. While the GFI was slightly below the ideal threshold at 0.850, the other indices, including AGFI, NFI, CFI, and TLI, exhibited values ranging from 0.937 to 0.960, affirming a commendable model fit (Table 5). The revised model, offering a visual representation of these findings, is illustrated in Fig. 1.

**Table 5:** Model fit indices of confirmatory factor analysis for PROMIS-29 V2.1 (n = 354).

Indices	χ^2^/df	RMSEA	GFI	CFI	IFI	NFI	TLI
PROMIS-29 V2.1	2.602	0.067	**0.850**	0.960	0.960	0.937	0.954
Criteria	< 3	< 0.08	> 0.9	> 0.9	> 0.9	> 0.9	> 0.9

*χ*^*2*^*/df* χ^2^/degree of freedom, *RMSEA* root mean square error of approximation, *GFI* comparative fit index, *CFI* comparative fit index, *IFI* incremental fit index, *NFI* Normed Fit Index, *TLI* Tucker–Lewis index, Bold = not meets cutoff.

### Convergent validity

The Average Variance Extracted (AVE) is the sum of the square of factor load, which represents the comprehensive explanation ability of the potential variable to all the measured variables. According to the general theory, the larger the AVE value, the stronger the potential variable's ability to explain its corresponding item at the same time; conversely, the stronger the item's ability to express the properties of the potential variable. When AVE > 0.5, convergent validity is good^15^, and when between 0.36 and 0.5, it is an acceptable range^15^. In this study, the AVE values for the seven dimensions of PROMIS-29 V2.1 range from 0.500 to 0.910. Each domain’s factor loadings, which are indicative of the relationships between the items and their respective constructs, were notably high across most domains, further corroborating this assertion, showing satisfactory convergent validity. See Table 6.

**Table 6:** The convergent validity of the PROMIS-29 V2.1

Path		Factor loading	S.E.	CR	AVE
Q211	Physical function	0.890		0.954	0.838
Q212	Physical function	0.912	0.038		
Q213	Physical function	0.910	0.037		
Q214	Physical function	0.948	0.038		
Q221	Anxiety	0.931		0.965	0.872
Q222	Anxiety	0.942	0.029		
Q223	Anxiety	0.948	0.028		
Q224	Anxiety	0.913	0.031		
Q231	Depression	0.907		0.966	0.878
Q232	Depression	0.945	0.032		
Q233	Depression	0.971	0.03		
Q234	Depression	0.923	0.031		
Q241	Fatigue	0.929		0.967	0.879
Q242	Fatigue	0.919	0.033		
V243	Fatigue	0.940	0.029		
Q244	Fatigue	0.961	0.027		
Q251	Sleep disturbance	0.510		0.778	0.500
Q252	Sleep disturbance	0.356	0.131		
Q253	Sleep disturbance	0.872	0.182		
Q254	Sleep disturbance	0.918	0.184		
Q261	Ability to participate in social roles and activities	0.898		0.959	0.855
Q262	Ability to participate in social roles and activities	0.969	0.033		
Q263	Ability to participate in social roles and activities	0.937	0.035		
Q264	Ability to participate in social roles and activities	0.893	0.038		
Q271	Pain interference	0.910		0.976	0.910
Q272	Pain interference	0.980	0.032		
Q273	Pain interference	0.979	0.033		
Q274	Pain interference	0.944	0.037		

*S.E.* standard error, *CR* Composite Reliability, *AVE* Average Variance Extracted.

### Discrimination validity

In this study, the seven dimensions of PROMIS-29 V2.1 were significantly correlated (p < 0.01), and the absolute correlation coefficients are all smaller than the corresponding √AVE, indicating that there is a certain correlation among all latent variables, and a certain degree of differentiation between each other, showing ideal discrimination validity. See Table 7.

**Table 7:** The discrimination validity of the PROMIS-29 V2.1

	Physical function	Anxiety	Depression	Fatigue	Sleep disturbance	Ability to participate in social roles and activities	pain interference
Physical function	1.000						
Anxiety	− 0.560**	1.000					
Depression	− 0.471**	0.900**	1.000				
Fatigue	− 0.660**	0.704**	0.702**	1.000			
Sleep disturbance	− 0.346**	0.529**	0.535**	0.570**	1.000		
Ability to participate in social roles and activities	0.624**	− 0.615**	− 0.612**	− 0.704**	− 0.559**	1.000	
pain interference	− 0.501**	0.544**	0.505**	0.617**	0.486**	− 0.558**	1.000
AVE	0.838	0.872	0.878	0.879	0.498	0.855	0.909
√AVE	0.915	0.934	0.937	0.937	0.705	0.925	0.954

**P < 0.01, *AVE* Average Variance Extracted, *√AVE* Average Variance Extracted square root.

## Discussion

This study is pioneering in its endeavor to evaluate the psychometric properties of the Chinese version of the PROMIS-29 V2.1 profile among patients with HM. Our findings affirm the reliability and validity of this instrument in capturing the multifaceted health status, encompassing physical, mental, and social dimensions, of this specific patient group.

Regarding reliability, Cronbach’s alpha is considered an adequate measure of internal consistency^16^. Composite Reliability (CR) reflects whether all questions in each latent variable consistently explain the latent variable, and when the value is higher than 0.70, it indicates that the latent variable has good CR^17^. Compared to Cronbach’s α, CR is more able to incorporate the different factor loadings of each observation item on latent variables into the calculation formula, and its estimated value is closer to the internal consistency reliability of the scale^17^. In this study, both the Cronbach’s α and CR of all domains were close to, or meeting the more stringent criterion of 0.9, which providing evidence of high internal consistency reliability.

The T-scores derived from the PROMIS-29 V2.1 highlighted an apparent diminution in physical function and social participation compared to the reference group. This underscores a pronounced impairment in physical activities and social engagement. The results were similar to those of patients with breast cancer^2^ and systemic sclerosis^18^.

Evidence of floor and ceiling effects has been observed in some PROMIS-29 V2.1 domains, which has also been noted in other PROMIS validation projects^8,19^. The floor and ceiling effects of the scale mean that the number of respondents who achieved the worst or the best possible score, which reflect the quantity scale features of score distribution^16^. Floor or ceiling effects are considered to be present if more than 15% of respondents achieved the worst or the best possible score, respectively^16,20^.

In our study, a significant proportion of participants reported minimal symptoms in anxiety, depression, fatigue, and pain domains, aligning with general population trends. However, pronounced ceiling effects in each domains (except sleep disturbance) could be attributed to the fact that a majority of our sample were undergoing treatment, potentially amplifying these effects. Nevertheless, it would not be problematic when identifying those with poor physical performance. Such limitations may not exist in a future sample including more patients at different stage of the disease.

The criterion validity was demonstrated by its varying degree of correlations with FACT-G. Criterion validity refers to the extent to which scores on a particular instrument relate to a gold standard^16^. Current studies on PROs or QOL in people with HM usually use the FACT-G as the assessment tool^21,22^. Spearman correlation coefficients > 0.50 were considered strong correlation, 0.30–0.50 indicated moderate correlation, and < 0.30 indicated weak correlation^15^. In this study, the PROMIS-29 V2.1 domains showed adequate correlations with all corresponding dimensions of the FACT-G (P < 0.01).

CFA showed that the Chinese version of the PROMIS-29 V2.1 in patients with HM had good evidence for construct validity including the presence of the seven domains. According to the results of goodness-of-fit, the model is considered to have a good fitting effect when χ^2^/df < 3, IFI > 0.9, and RMSEA < 0.08 after correction, meanwhile, the values of the five fitting indices (GFI, AGFI, NFI, CFI, and TLI) should be all between 0 and 1, the closer to 0, the worse the fitting, and the closer to 1, the better the fitting^15^. The goodness-of-fit indices for the original domain of PROMIS-29 V2.1 were high. Meanwhile, the PROMIS-29 V2.1 were showing satisfactory convergent validity and discrimination validity. The results underscore the robust structural integrity of the PROMIS-29 V2.1 in capturing the multifaceted health outcomes of patients with HM.

Convergent validity which is evaluated by the AVE index, means that items measuring the same underlying domain should belong to the same dimension and there should have a high degree of correlation between items^15^. In the context of this study, the AVE values for all seven domains of the PROMIS-29 V2.1 were examined, offering insights into the measure’s convergent validity among patients with HM. These findings underscore the instrument’s robustness in capturing the intended constructs with minimal measurement error, attesting to its utility in this specific patient population. The consistency in factor loadings amplifies confidence in the PROMIS-29 V2.1’s ability to offer reliable, nuanced insights into the multifaceted health outcomes of patients with HM.

Discriminant validity evaluates the extent to which a construct is distinct from other constructs, ensuring that it is not highly correlated with other variables, and should theoretically be different from^15^. In this context, it is assessed by comparing the√AVE for each construct with the correlations between that construct and others. Ideal discriminant validity is achieved when the √AVE for each construct is greater than its highest correlation with any other construct^15^. In our study, the PROMIS-29 V2.1 demonstrated excellent discriminant validity among patients with hematologic malignancies. For instance, while there was a notable correlation between anxiety and depression (r = 0.900, p < 0.01), the √AVE values for these constructs were 0.934 and 0.937, respectively, exceeding the correlation coefficient. This pattern was consistent across all construct pairs, underscoring the instrument’s ability to distinguish between different aspects of patients’ health and well-being effectively. These findings affirm the multidimensionality of the PROMIS-29 V2.1 and its applicability in capturing a broad spectrum of health outcomes among patients with hematologic malignancies, without conflating distinct constructs.

To sum up, these findings reinforce the utility of the Chinese version of the PROMIS-29 V2.1 as a reliable tool, mirroring the intricate nuances of patients’ experiences and outcomes. This congruence in outcomes underscores the PROMIS-29 V2.1’s potential as a pivotal tool in both clinical and research settings for this patient population.

### Limitations

However, this study has several limitations. First, the participant pool, though multicentric, was confined to tertiary hospitals in China, warranting caution in extrapolating these findings to broader settings and populations. Second, the cross-sectional design precludes insights into the instrument’s responsiveness and interpretability over varying clinical states, marking an avenue for future longitudinal studies. Third, this study doesn’t explain how the questionnaires work in the pre- and post-treatment patient population, and that's what we’re going to explore next.

## Conclusion

This study meticulously evaluated the psychometric properties of the Chinese version of the PROMIS-29 V2.1 in patients with HM, utilizing a comprehensive, multicenter sample. Our findings affirm that this version of PROMIS-29 V2.1 is a validated and reliable instrument, adept at measuring a spectrum of symptoms and functional attributes in HM patients. However, the evolution of this instrument’s applicability doesn’t end here. Future studies should consider incorporating Item Response Theory (IRT) methodologies. This advanced approach will facilitate a nuanced, micro-level analysis of item performance, enhancing the precision and applicability of the instrument. In conclusion, our study not only underscores the psychometric properties of the Chinese version of the PROMIS-29 V2.1 but also paves the way for its widespread adoption in assessing and monitoring symptoms and functions among Chinese patients with HM.

## Data Availability

All data generated or analyzed during this study are included in this published article.
